# E-Cigarette Promotion on Twitter in Australia: Content Analysis of Tweets

**DOI:** 10.2196/15577

**Published:** 2020-11-05

**Authors:** Kahlia McCausland, Bruce Maycock, Tama Leaver, Katharina Wolf, Becky Freeman, Katie Thomson, Jonine Jancey

**Affiliations:** 1 Collaboration for Evidence, Research and Impact in Public Health School of Public Health Curtin University Bentley Australia; 2 College of Medicine and Health University of Exeter Devon United Kingdom; 3 School of Media, Creative Arts and Social Inquiry Curtin University Bentley Australia; 4 School of Marketing Curtin University Bentley Australia; 5 School of Public Health University of Sydney Sydney Australia; 6 School of Public Health Curtin University Bentley Australia

**Keywords:** electronic cigarette, e-cigarette, electronic nicotine delivery systems, vaping, vape, social media, twitter, content analysis, public health, public policy

## Abstract

**Background:**

The sale of electronic cigarettes (e-cigarettes) containing nicotine is prohibited in all Australian states and territories; yet, the growing availability and convenience of the internet enable the promotion and exposure of e-cigarettes across countries. Social media’s increasing pervasiveness has provided a powerful avenue to market products and influence social norms and risk behaviors. At present, there is no evidence of how e-cigarettes and vaping are promoted on social media in Australia.

**Objective:**

This study aimed to investigate how e-cigarettes are portrayed and promoted on Twitter through a content analysis of vaping-related tweets containing an image posted and retweeted by Australian users and how the portrayal and promotion have emerged and trended over time.

**Methods:**

In total, we analyzed 1303 tweets and accompanying images from 2012, 2014, 2016, and 2018 collected through the Tracking Infrastructure for Social Media Analysis (TrISMA), a contemporary technical and organizational infrastructure for the tracking of public communication by Australian users of social media, via a list of 15 popular e-cigarette–related terms.

**Results:**

Despite Australia’s cautious approach toward e-cigarettes and the limited evidence supporting them as an efficacious smoking cessation aid, it is evident that there is a concerted effort by some Twitter users to promote these devices as a health-conducive (91/129, 70.5%), smoking cessation product (266/1303, 20.41%). Further, Twitter is being used in an attempt to circumvent Australian regulation and advocate for a more liberal approach to personal vaporizers (90/1303, 6.90%). A sizeable proportion of posts was dedicated to selling or promoting vape products (347/1303, 26.63%), and 19.95% (260/1303) were found to be business listings. These posts used methods to try and expand their clientele further than immediate followers by touting competitions and giveaways, with those wanting to enter having to perform a sequence of steps such as liking, tagging, and reposting, ultimately exposing the post among the user’s network and to others not necessarily interested in vaping.

**Conclusions:**

The borderless nature of social media presents a clear challenge for enforcing Article 13 of the World Health Organization Framework Convention on Tobacco Control, which requires all ratifying nations to implement a ban on tobacco advertising, promotion, and sponsorship. Countering the advertising and promotion of these products is a public health challenge that will require cross-border cooperation with other World Health Organization Framework Convention on Tobacco Control parties. Further research aimed at developing strategies to counter the advertising and promotion of e-cigarettes is therefore needed.

## Introduction

In Australia, the context of the present study, the legal status of electronic cigarettes (e-cigarettes) is determined by existing and overlapping laws relating to poisons, therapeutic and consumer goods, and tobacco control [1]. Liquid nicotine is classified as a “Schedule 7-Dangerous Poison” under the Federal Poisons Standard [2], and, as such, the manufacture, sale, or supply of e-cigarettes containing nicotine without lawful authority (ie, prescription from a medical doctor) [3] is prohibited in all Australian states and territories [4]. However, nicotine-containing e-cigarettes can be imported into Australia, as there is no way to determine whether or not an e-cigarette contains nicotine, short of laboratory analysis, which has implications for law enforcement [4,5]. E-cigarettes that do not contain nicotine can be sold in some Australian jurisdictions, provided manufacturers do not make therapeutic claims, while the sale and use of flavored e-liquid are permitted provided it does not contain nicotine [4].

The World Health Organization Framework Convention on Tobacco Control (WHO FCTC) defines tobacco advertising and promotion as “any form of commercial communication, recommendation or action with the aim, effect or likely effect of promoting a tobacco product or tobacco use either directly or indirectly” and requires signatories to the treaty, of which Australia is one, to “undertake a comprehensive ban on all tobacco advertising, promotion and sponsorship” [6]. As nicotine-containing e-cigarettes are banned from retail sale in Australia; the advertising of such products is also not permitted. Further, advertising of all types of e-cigarette products and devices, nonnicotine included, is regulated at the state level, with most states prohibiting any form of advertising or promotion [7-10].

Data from the most recent National Drug Strategy Household Survey [11] report 11.3% of Australians aged over 14 years have ever used, and 2.5% currently use, e-cigarettes, increasing from 8.8% and 1.2%, respectively, since 2016. These increases occurred in both smokers and nonsmokers and contrast with Australian combustible smoking rates, which have continued to decline over the last 30 years. The most frequent reason for using e-cigarettes reported by people older than 14 years was “out of curiosity” (54.2%). Further, 22.8% cited using e-cigarettes because they perceived them to be less harmful than tobacco cigarettes (19.2% in 2016), and 10.1% believed vaping to be more socially acceptable than tobacco smoking (6.0% in 2016). In addition, 26.9% of respondents reported they obtained their e-cigarette products online (Australian retailer 12.5%, overseas retailer 11.1%, unknown origin 3.3%).

Vaping has become increasingly popular, and awareness, experimentation, and uptake have proliferated both within Australia and globally [12]. Researchers have therefore begun harnessing data from social media to address information gaps, provide timely insights, and inform public policy and public health [13-15]. As of January 2019, there were approximately 2.56 million active monthly Australian Twitter users (64% male), which equates to approximately 12% of Australians older than 13 years [16]. Given the popularity of Twitter [16], the high-speed nature of information dissemination, and the significant influence of Twitter as a driver of web traffic [17], insights into how Twitter is used to promote and discuss e-cigarettes are warranted.

Social media’s increasing pervasiveness has provided a powerful avenue to market products and influence social norms and behaviors [18]. There is mounting evidence of the volume of e-cigarette promotion on social media [19,20], with studies suggesting adolescents who view e-cigarette social media promotion express greater intention to use e-cigarettes, more positive attitudes toward e-cigarettes, and greater perceptions of e-cigarette use as normative [21,22]. This is concerning, as Australia’s current regulatory stance has proven effective in limiting e-cigarette uptake [11]; however, promotion on social media could bring awareness to and encourage experimentation with e-cigarettes or other tobacco products [23,24]. The health effects of e-cigarette use are not fully understood; however, a growing body of literature has established acute consequences with even short-term use, with [25] or without nicotine [26,27].

A 2019 scoping review [19] that aimed to identify and describe the messages presented in e-cigarette–related social media promotions and discussions across the United Kingdom, United States, New Zealand, Canada, and Australia identified no studies from Australia. At the time of this study, there was no published literature on how e-cigarettes are promoted and discussed online in the Australian context. We, therefore, aimed to investigate how e-cigarettes are portrayed and promoted on Twitter through a content analysis of related tweets posted and retweeted by Australian users and how the portrayal and promotion have trended over time in the Australian context where e-cigarettes are largely prohibited.

## Methods

### Data Collection

Twitter data were collected via Tracking Infrastructure for Social Media Analysis (TrISMA) [28], a contemporary technical and organizational infrastructure for the tracking of public communication by Australian users of social media. Central to the TrISMA Twitter infrastructure is the Australian Twitter Collection, which continuously gathers tweets from identified Australian accounts (ie, accounts set to an Australian location, geolocation, or time zone or accounts with a description field referring to an Australian location or containing Australia-specific terms) and stores them in a database available to accredited TrISMA researchers. The TrISMA Twitter Collection is hosted on a cloud-based Google BigQuery database and accessed through the data visualization tool Tableau.

A list of popular e-cigarette–related terms was developed based on peer-reviewed literature [29-34], trending Twitter hashtags, and frequently co-occurring hashtags (ie, hashtags that appeared in the same caption as the root term), which resulted in 15 keywords: *cloudchasing, ecig* (includes ecigarette/s), *e-cig* (includes e-cigarette/s), *electroniccig* (includes electroniccigarette/s), *electronic cigarette* (includes electronic cigarettes), *eliquid, e-liquid, e-juice, vape* (includes vaper and vapes), *vaping, vapecommunity, vapefam, vapelife, vapenation*, and *vapeporn*. E-cigarette product names were omitted from the search strategy so as not to bias the results to specific brands [35]. A preliminary search revealed there was minimal Twitter activity using these keywords before 2012. Therefore, 2 yearly sampling intervals starting from 2012 to 2018 were chosen to maximize the period of time covered while still being able to see the emergence and decline of trends in the collected data.

Data (tweets), along with meta-data information (ie, username, user follower count), were collected from public Australian Twitter users when a tweet included at least one of the identified keywords from either respective year. Data were downloaded in the form of comma-separated values files for each keyword and respective year. Social media users tend to include multiple hashtags within their posts, which resulted in duplicate tweets being collected. Duplicate tweets within keyword corpora for each year and across keyword corpora from the co-use of hashtags were removed, resulting in the inclusion of only unique tweets [36].

Data were assigned a number in ascending order, and 100 tweets from each keyword corpus for each year were randomly selected using an online random sequence generator [37]. Selected data were checked by one researcher (KM) to determine eligibility (ie, written in English and relevant to e-cigarettes). If any of the originally selected 100 tweets did not fit the inclusion criteria, further sampling occurred until 100 eligible tweets were reached. If a keyword corpus had less than 100 tweets, then all eligible tweets were selected. Each tweet was inspected, and, if found to contain an image, a screenshot of the whole post (text and image) was saved for further analysis. Eligible images needed to be stationary (ie, not a video, animated graphic interchange format [GIF], or other moving content). Only posts that contained an image were included in this study as the influence of the “picture superiority effect,” which specifies pictures and images are more likely to be remembered than words, is widely acknowledged [38]. Social media content that includes associated imagery is also more noticeable, shareable, and engaging to users [39].

Retweets (tweets reposted by users) were included in this study, which facilitated the understanding of what information was being circulated by Australian users, even if it originated in another country.

### Ethical Considerations

A particularly salient concern among researchers is whether social media data should be considered public or private data [40]. Twitter is a social networking service in which users broadcast their opinions and commonly use a hashtag to associate their thoughts on a subject with users on the same subject; therefore, this data is generally referred to as “public data” [40]. For ethical, privacy, and technical reasons, TrISMA does not collect tweets from private accounts or direct messages; therefore, all data collected in this study were publicly available. This study was approved by the Curtin University Human Research Ethics Committee (approval number: HRE2017-0144).

### Developing the Coding Framework

A concept-driven approach (inductive) [41] informed by extant studies [29-34] was utilized to develop a coding framework. The coding frame was tested on a random sample of 100 tweets by 2 researchers (KM and KT), whereby each tweet was read and assigned codes based upon the concepts presented in the descriptive text, hashtags, and accompanying image [42]. It is critical to consider the visual and textual aspects of posts together in the analysis [42] as the study of images can be used to complement and extend the study of health behaviors and may be more valuable than the study of words alone [15]. The 2 researchers followed a hybrid inductive/deductive content analysis approach [41] to refine and further develop the coding framework before transferring the modified framework into IBM SPSS Statistics (v22).

### Interrater Reliability Testing

The 2 researchers applied the modified coding framework to a sample of 140 randomly selected posts (approximately 10% of the final sample), and an interrater reliability test was performed. Interrater reliability was determined using Krippendorff alpha, and an average score of α=.89 was obtained, with a range of .65-1.0, indicating good to perfect agreement [43]. Any discrepancies were discussed to reach consensus, and the coding framework was revised accordingly.

### Coding and Analysis

The final coding framework (Multimedia Appendix 1) was applied by KT and checked for consistency and validity by KM. The coders met regularly to refine coding rules and discuss questions and emergent themes. Each code within the coding framework was a variable in SPSS that functioned as a standalone item and was evaluated as either 1 for present or 2 for absent. Statistical comparisons (ie, between codes and years) were made using chi-square tests or Fisher exact tests, if applicable. Data were analyzed using IBM SPSS Statistics (v22). Due to the small sample size of the 2012 data, a further sensitivity analysis was performed with statistical comparisons made using chi-square and Fisher exact tests to assess the robustness of the results by removing the observations in 2012.

## Results

### Sample of Posts

Of the 4437 randomly selected tweets, 1553 contained an image, and an eligible sample of 1303 tweets was retained for analysis (Table 1).

**Table 1:** Number of posts selected for analysis.

Year of post	Random sample of posts (n=4437), n	Posts containing an image (n=1553), n	Posts eligible for analysis (n=1303), n
2012	570	12	12
2014	1,196	289	246
2016	1,378	658	540
2018	1,293	594	505

### Sensitivity Analysis

After performing the sensitivity analysis, all associations, except for one, remained significant when removing the 12 observations from 2012. After the removal of the 2012 data, the “quit smoking” association did not retain its significance (*P*=.213). The results of the sensitivity analysis indicate that, overall, the results were not substantially influenced by the small number of data in 2012.

#### Frequency and Description of Codes

##### Overview

In total, 1303 tweets and accompanying images (collectively referred to as posts) were analyzed: 12 from 2012, 246 from 2014, 540 from 2016, and 505 from 2018.

##### People

Of the images that contained a person, 60.0% (326/543) portrayed a man, and the majority of people appeared to be over the age of 18 years (300/313, 95.8%; Table 2). The largest proportion of people visible in these images was classified as “everyday people” (283/543, 52.1%).

**Table 2:** Frequency statistics for each year corpus and the total sample within the “people” domain.

Associated codes		2012 (N=12), n (%)		2014 (N=246), n (%)		2016 (N=540), n (%)		2018 (N=505), n (%)		Total (N=1303), n (%)	
People visible		4 (33.3)		115 (46.7)		209 (38.7)		215 (42.6)		543 (41.7)	
**Type of people visible**											
	Everyday person		2 (50.0)^a^		65 (56.5)^b^		120 (57.4)^c^		96 (44.6)^d^		283 (52.1)^e^
	Model		1 (25.0)^a^		39 (33.9)^b^		59 (28.2)^c^		78 (36.3)^d^		177 (32.6)^e^
	Celebrity		1 (25.0)^a^		4 (3.5)^b^		9 (4.3)^c^		15 (7.0)^d^		29 (5.3)^e^
	Health professional/academic		0 (0)^a^		0 (0)^b^		3 (1.4)^c^		12 (5.6)^d^		15 (2.8)^e^
	Other		0 (0)^a^		7 (6.1)^b^		11 (5.3)^c^		4 (1.9)^d^		22 (4.1)^e^
	Multiple types		0 (0)^a^		0 (0)^b^		7 (3.3)^c^		10 (4.6)^d^		17 (3.1)^e^
**Gender of people visible**											
	Female		1 (25.0)^a^		39 (33.9)^b^		44 (21.0)^c^		39 (18.1)^d^		123 (22.7)^e^
	Male		3 (75.0)^a^		58 (50.4)^b^		134 (64.1)^c^		131 (60.9)^d^		326 (60.0)^e^
	Both		0 (0)^a^		7 (6.1)^b^		15 (7.2)^c^		23 (10.7)^d^		45 (8.3)^e^
	Cannot determine		0 (0)^a^		11 (9.6)^b^		16 (7.7)^c^		22 (10.2)^d^		49 (9.0)^e^
**Age of people visible (years)**											
	<18		0 (0)^f^		0 (0)^g^		3 (2.6)^h^		4 (3.2)^i^		7 (2.2)^j^
	≥18		2 (100.0)^f^		72 (100.0)^g^		111 (95.7)^h^		115 (93.5)^i^		300 (95.8)^j^
	Mixed		0 (0)^f^		0 (0)^g^		2 (1.7)^h^		4 (3.2)^i^		6 (1.9)^j^

^a^N=4.

^b^N=115.

^c^N=209.

^d^N=215.

^e^N=543.

^f^N=2.

^g^N=72.

^h^N=116.

^i^N=123.

^j^N=313.

##### Product Placement and Visibility

A vaporizer product was visible in 70% (913/1303) of images, and most commonly (497/1303, 38.14%) these were e-cigarette or other vaping devices (eg, e-hookah, e-cigar; Table 3). E-cigarette liquids (also known as e-liquid or e-juice) were present in 11.82% (154/1303) of images. In posts that depicted a vaporizer product, the product was placed overtly within the image in 92.7% (846/913) of posts.

**Table 3:** Frequency statistics for each year corpus and the total sample within the “vape and tobacco products” domain.

Associated codes		2012 (N=12), n (%)		2014 (N=246), n (%)		2016 (N=540), n (%)		2018 (N=505), n (%)		Total (N=1303), n (%)	
**Product placement^a^**											
	Overt		8 (88.9)^b^		179 (94.2)^c^		373 (93.5)^d^		286 (90.8)^e^		846 (92.7)^f^
	Covert		1 (11.1)^b^		11 (5.8)^c^		26 (6.5)^d^		29 (9.2)^e^		67 (7.3)^f^
**Product visible**											
	E-cigarette or another vaping device		3 (25.0)^b^		116 (47.2)^c^		199 (36.9)^d^		149 (35.4)^e^		497 (38.1)^f^
	E-cigarette and another vape/tobacco product		2 (16.7)^b^		37 (15.0)^c^		79 (14.6)^d^		37 (7.3)^e^		155 (11.9)^f^
	Vape accessory		0 (0)^b^		11 (4.5)^c^		28 (5.2)^d^		22 (4.4)^e^		61 (4.7)^f^
	Vape liquid (e-liquid)		1 (8.3)^b^		17 (6.9)^c^		84 (15.6)^d^		52 (10.3)^e^		154 (11.8)^f^
	Vape liquid and another vape/tobacco product		1 (8.0)^b^		0 (0)^c^		0 (0)^d^		5 (1.0)^e^		6 (0.5)^f^
	Showcase in a retail store		0 (0)^b^		6 (2.4)^c^		4 (0.7)^d^		5 (1.0)^e^		15 (1.2)^f^
	Tobacco product		2 (16.7)^b^		3 (1.2)^c^		4 (0.7)^d^		14 (2.8)^e^		23 (1.8)^f^
**Setting**											
	Indoors		4 (66.7)^g^		94 (77.7)^h^		173 (71.2)^i^		107 (60.1)^j^		378 (69.0)^k^
	Outdoors		2 (33.3)^g^		27 (22.3)^h^		70 (28.8)^i^		71 (39.9)^j^		170 (31.0)^k^

^a^Only coded for if a product was visible in the post.

^b^N=9.

^c^N=190.

^d^N=399.

^e^N=315.

^f^N=913

^g^N=6.

^h^N=121.

^i^N=243.

^j^N=178.

^k^N=548.

##### Promotional Practices and Strategies

**Figure 1:**
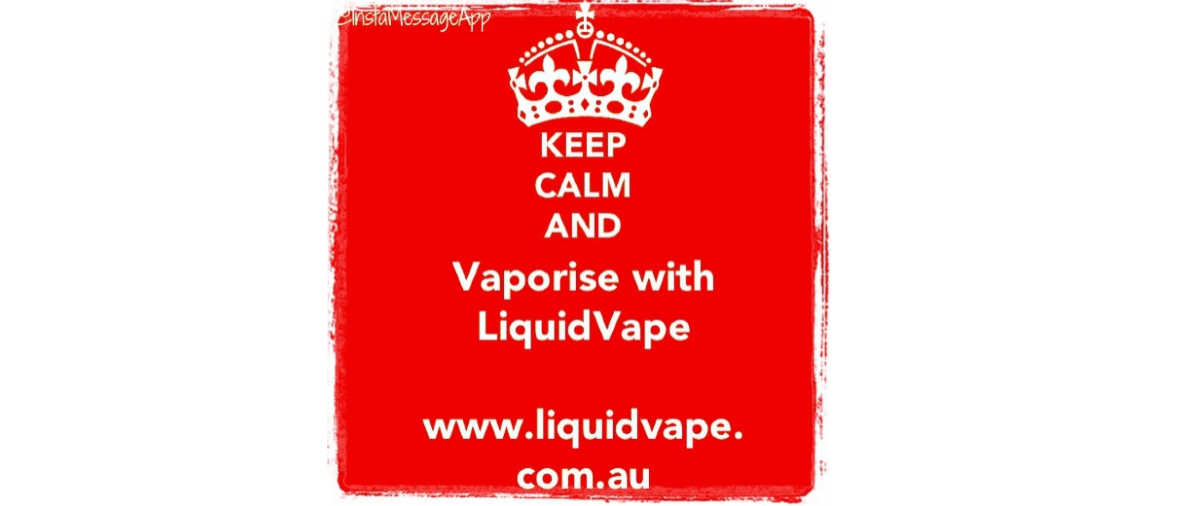
Example within the business listing category of the "promotional practices and strategies" domain.

**Figure 2:**
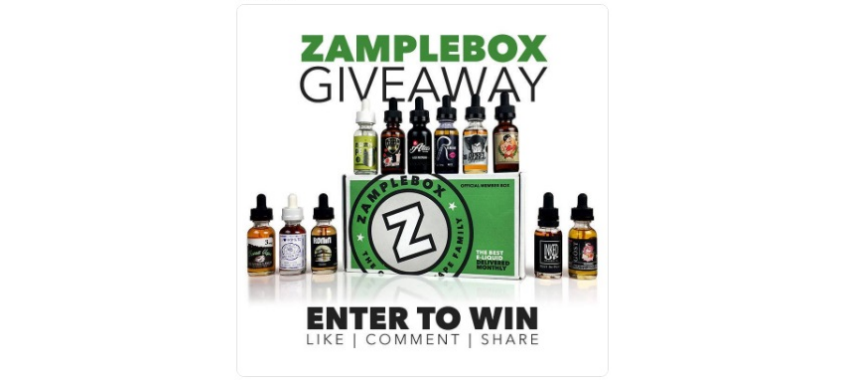
Example within the nonmonetary promotional offer category of the "promotional practices and strategies" domain.

In 26.63% (347/1303) of posts, purchase of e-cigarette products was promoted, and 9.67% (126/1303) of posts provided Twitter users with a promotional offer (Table 4). Promotional offers could be monetary or nonmonetary, of which nonmonetary offers were most prevalent (86/126, 68.3%). Nonmonetary promotional offers did not lower the cost of a purchase; they instead promoted contests, giveaways, and sweepstakes or offered free shipping or a free gift with purchase. Rather than aiming to sell specific e-cigarette products, some posts promoted vape businesses, brands, and online groups. These posts were categorized as “business listings” and comprised 19.95% (260/1303) of the total sample (Figure 1). Some business listings and promotional posts used methods to increase their visibility and expand their market, such as operating competitions to win e-cigarette products. However, to enter a competition, Twitter users were required to undertake a series of steps including following the account, and liking, commenting, re-tweeting, or tagging others in the post (Figure 2).

**Figure 3:**
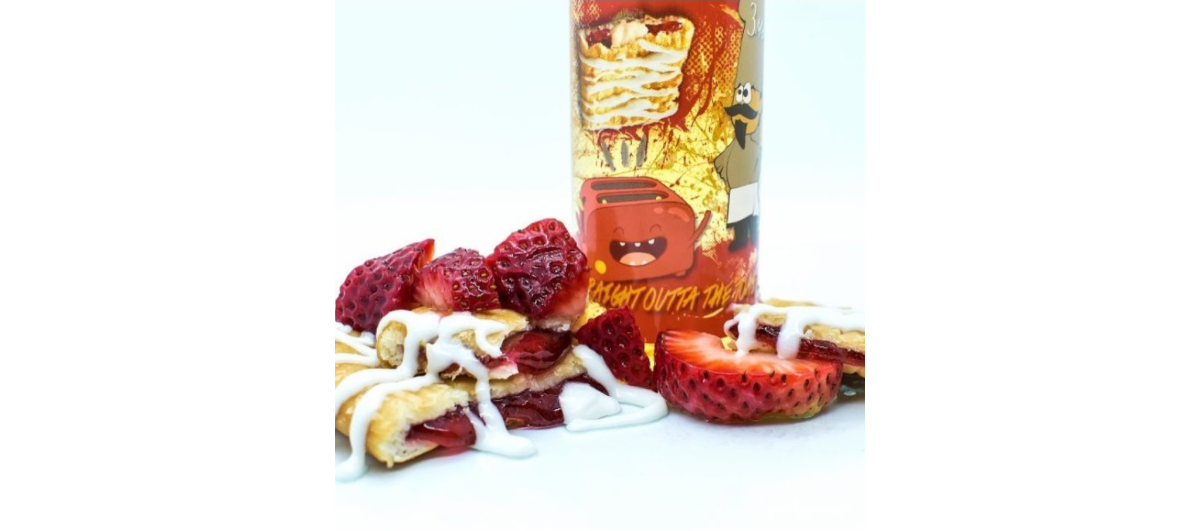
Example of a flavor within the "promotional practices and strategies" domain.

Of posts that displayed or discussed e-liquid products, 71.1% (226/318) described the flavor of the product through either words or images (eg, images of candy or fruits; Figure 3). Creative flavor names (eg, King Cookie Dough, Show me the Honey) and descriptive flavor descriptions (eg, “Grab a sweet and spicy cup of tea from the Chai Wallah as he makes the rounds on an overcrowded train slowly making its way to Varanasi”) were commonly depicted in image captions and on product packaging.

**Table 4:** Frequency statistics for each year corpus and the total sample within the “promotional practices and strategies” domain.

Associated codes		2012 (N=12), n (%)		2014 (N=246), n (%)		2016 (N=540), n (%)		2018 (N=505), n (%)		Total (N=1303), n (%)	
E-liquid flavor described (yes)^a^		3 (100.0)^b^		33 (58.9)^c^		144 90.0)^d^		76 (76.8)^e^		226 (71.1)^f^	
Product brand or logo visible (yes)^g^		4 (44.4)^h^		83 (43.7)^i^		230 (57.6)^j^		144 (45.7)^k^		461 (50.5)^l^	
Product brand or logo is visible anywhere		4 (33.3)		128 (52.0)		275 (50.9)		211 (41.8)		618 (47.4)	
Promoting vape product for purchase		2 (16.7)		80 (32.5)		164 (30.4)		101 (20.0)		347 (26.6)	
Business listing		2 (16.7)		61 (24.8)		101 (18.7)		96 (19.0)		260 (20.0)	
Vapor present		1 (8.3)		60 (24.4)		104 (19.3)		89 (17.6)		254 (19.5)	
**Promotional offer**											
	Monetary		0 (0)^m^		11 (42.3)^n^		15 (31.9)^o^		7 (7.8)^p^		33 (26.2)^q^
	Nonmonetary		1 (100.0)^m^		14 (53.8)^n^		29 (61.7)^o^		45 (86.5)^p^		89 (70.6)^q^
	Both		0 (0)^m^		1 (3.8)^n^		3 (6.4)^o^		0 (0.0)^p^		4 (3.2)^q^
Vape product review		0 (0)		7 (2.8)		36 (6.7)		29 (5.7)		72 (5.5)	
Cartoon		1 (8.3)		8 (3.3)		31 (5.7)		18 (3.6)		58 (4.5)	
Sale notice		0 (0)		3 (1.2)		11 (2.0)		1 (0.2)		15 (1.2)	

^a^Only coded for if the post displayed or discussed an e-liquid product.

^b^N=3.

^c^N=56.

^d^N=160.

^e^N=99.

^f^N=318.

^g^Only coded for if a vaping-related product was visible in the post.

^h^N=9.

^i^N=190.

^j^N=399.

^k^N=315.

^l^N=913.

^m^N=1.

^n^N=26.

^o^N=47.

^p^N=52.

^q^N=126.

##### Health, Safety, and Product Claims

**Figure 4:**
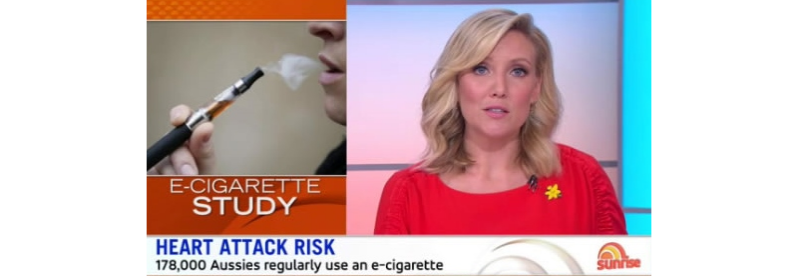
Example of health consequences being explained within the “health, safety, and product claims” domain.

**Figure 5:**
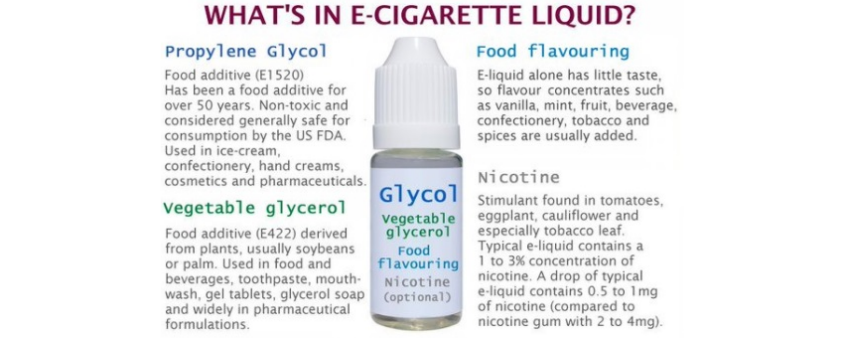
Example of an explanation of e-liquid ingredients within the “health, safety, and product claims” domain.

**Figure 6:**
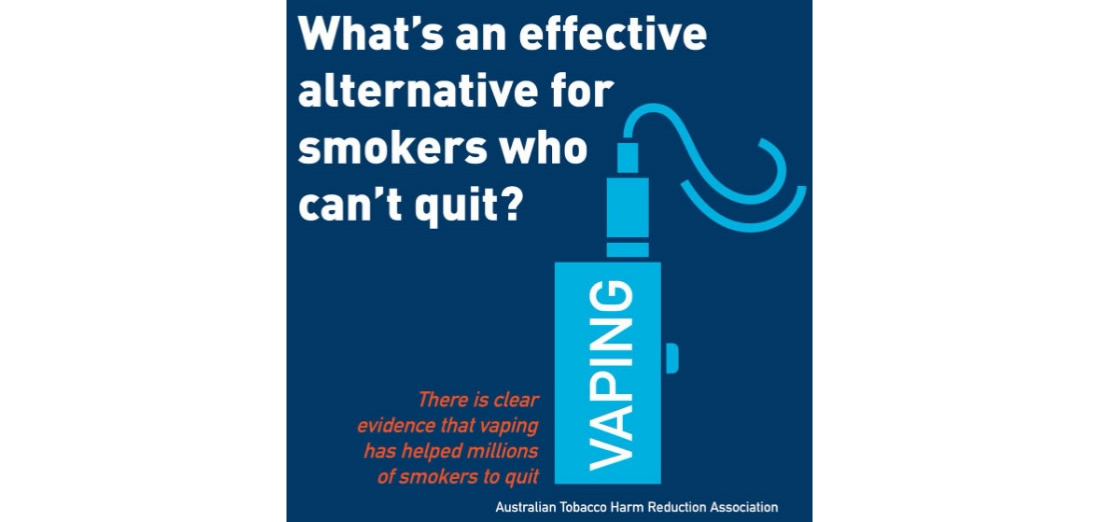
Example of describing e-cigarettes as a smoking cessation aid within the “health, safety, and product claims” domain.

The potential health benefits and consequences (Figure 4) of e-cigarettes were detailed in 9.90% (129/1303) of posts, of which 70.5% (91/129) conveyed the perceived benefits associated with e-cigarette use (Table 5). These posts compared e-cigarette products to their presumed more harmful counterpart, combustible cigarettes, by listing the alleged harmless ingredients found in vaporizer products (eg, nicotine, propylene glycol, glycerin, flavoring; Figure 5) compared to the toxic ingredients found in tobacco cigarettes (eg, ammonia, carbon monoxide, lead), labelled e-cigarettes as “smoke-free,” publicized that e-cigarettes provide a “safe” or “safer” smoking experience, and included testimonials from people who had quit smoking through the use of e-cigarettes and their subsequent positive changes in health. Further, a significant proportion of posts promoted e-cigarettes as an effective smoking cessation aid (266/1303, 20.41%; Figure 6).

**Table 5:** Frequency statistics for each year corpus and the total sample within the “health, safety, and product claims” domain.

Associated codes		2012 (N=12), n (%)		2014 (N=246), n (%)		2016 (N=540), n (%)		2018 (N=505), n (%)		Total (N=1303), n (%)	
Quit smoking		7 (58.3)		52 (21.1)		96 (17.8)		111 (22.0)		266 (20.4)	
**Health**											
	Total		1 (8.3)		15 (6.1)		34 (6.3)		79 (15.6)		129 (9.9)
	Positive		1 (100.0)		13 (86.7)		22 (64.7)		55 (69.6)		91 (75.2)
	Negative		0 (0)		2 (13.3)		12 (35.3)		24 (30.4)		38 (29.5)
Safety		0 (0)		8 (3.3)		30 (5.6)		24 (4.8)		62 (4.8)	
Public health		0 (0)		2 (0.8)		18 (3.3)		30 (5.9)		50 (3.8)	
Youth vaping		0 (0)		3 (1.2)		8 (1.5)		31 (6.1)		42 (3.2)	
Health warning or age restriction visible		0 (0)		3 (1.2)		8 (1.5)		14 (2.8)		25 (1.9)	
**Nicotine^a^**											
	Nicotine level (mg)		0 (0)^b^		4 (8.7)^c^		27 (18.1)^d^		3 (3.9)^e^		34 (12.4)^f^
	Nicotine-free		1 (50.0)^b^		1 (2.3)^c^		9 (6.0)^d^		9 (11.7)^e^		20 (7.3)^f^
	Multiple products: nicotine and nicotine-free		0 (0)^b^		2 (4.3)^c^		2 (1.3)^d^		1 (1.3)^e^		5 (1.8)^f^
	No nicotine level visible		1 (50.0)^b^		39 (84.8)^c^		111 (74.5)^d^		64 (83.1)^e^		215 (78.5)^f^

^a^Only coded for if the post displayed an e-liquid product.

^b^N=2.

^c^N=46.

^d^N=149.

^e^N=77.

^f^N=274.

**Figure 7:**
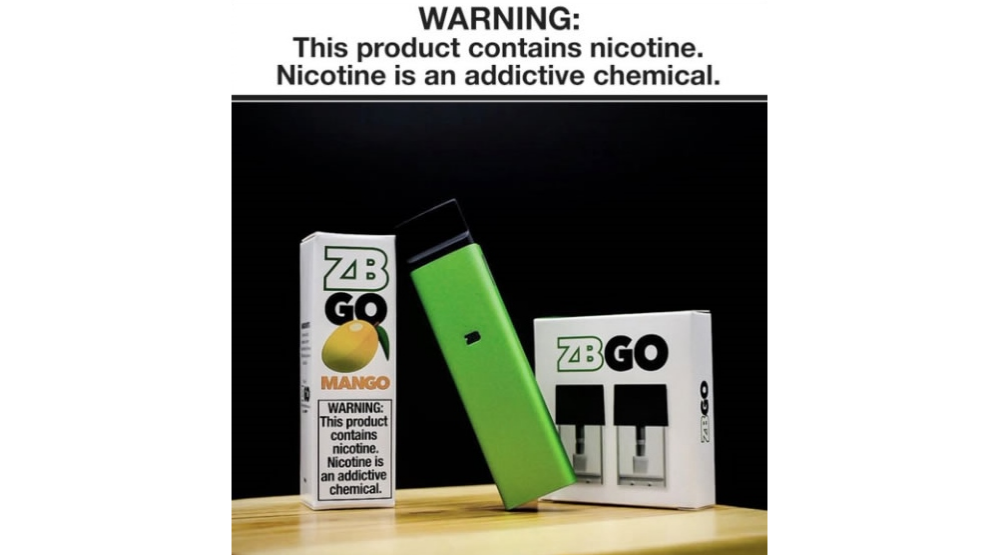
Example of a health warning within the “health, safety, and product claims” domain.

Only 1.92% (25/1303) of posts contained a health warning or age restriction. Health warnings were commonly displayed on e-cigarette product packaging (Figure 7). Age restrictions indicating products were not to be used by those under the age of 18 years were commonly asserted by a small icon, similar to that found on alcoholic beverages in Australia. Of the posts that portrayed an e-liquid product, 21.5% (59/274) identified whether the product contained nicotine (eg, 2 mg) or was nicotine-free (eg, 0 mg).

##### Behaviors and Practices

Over half (709/1303, 54.41%) of all posts indicated the presence of a vaping community or shared social identity or addiction bond, commonly through the use of hashtags. Popular hashtags that accompanied these posts included #vapecommunity, #vapefam, #vapenation, and #vapelife. One user posted:

#vape #vapefam #WeVapeWeVote #vapenation As a show of solidarity, I will add your #THR [tobacco harm reduction] medal to your profile pic[ture] if you’d like. Simply send me a DM [direct message] w/ [with] the picture and it can be done quickly.

**Figure 8:**
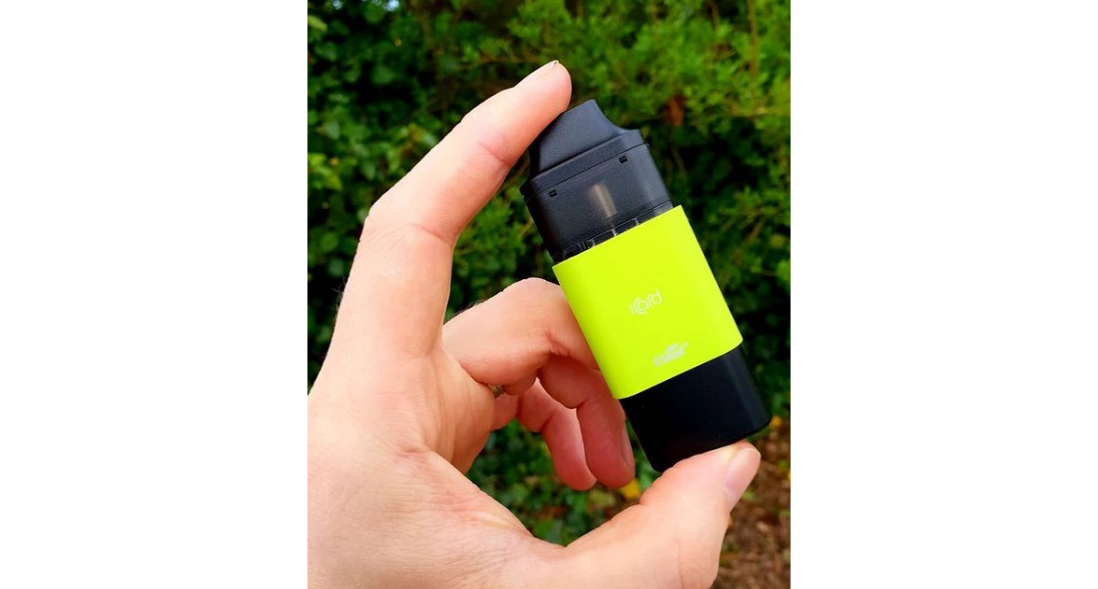
Example of a hand check post within the "behaviors and practices" domain.

“Hand check/product check” posts (255/1303, 19.57%) often appeared as simple photographs of an e-cigarette device or liquid in the hand of its user (Figure 8) or standalone (Table 6). These images were commonly taken in people’s homes, cars, and other outdoor locations and were frequently accompanied by the hashtag #handcheck.

**Table 6:** Frequency statistics for each year corpus and the total sample within the “behaviors and practices” and “association with another substance” domains.

Associated codes		2012 (N=12), n (%)	2014 (N=246), n (%)	2016 (N=540), n (%)	2018 (N=505), n (%)	Total (N=1303), n (%)
**Behaviors and practices domain**						
	Identity or community	0 (0)	142 (57.7)	341 (63.1)	226 (44.8)	709 (54.4)
	Hand check/product check	4 (33.3)	45 (18.3)	127 (23.5)	79 (15.6)	255 (19.6)
	Selfie	0 (0)	17 (6.9)	24 (4.4)	14 (2.8)	55 (4.2)
	Building/DIY^a^	1 (8.3)	10 (4.1)	21 (3.9)	18 (3.6)	50 (3.8)
	Meme	0 (0)	4 (1.6)	17 (3.1)	26 (5.1)	47 (3.6)
	Vape play	0 (0)	12 (4.9)	21 (3.9)	10 (2.0)	43 (3.3)
	Person vaping	1 (8.3)	71 (28.9)	99 (18.3)	90 (17.8)	261 (20.0)
	Erotic or sexualized	0 (0)	7 (2.8)	11 (2.0)	1 (0.2)	19 (1.5)
**Association with another** **substance domain**						
	Cannabis (including hemp)	0 (0)^b^	1 (25.0)^c^	11 (61.1)^d^	11 (91.7)^e^	23 (67.6)^f^
	Alcohol	0 (0)^b^	3 (75.0)^c^	7 (38.9)^d^	1 (8.3)^e^	11 (32.4)^f^

^a^DIY: do-it-yourself.

^b^N=0.

^c^N=4.

^d^N=18.

^e^N=12.

^f^N=34.

**Figure 9:**
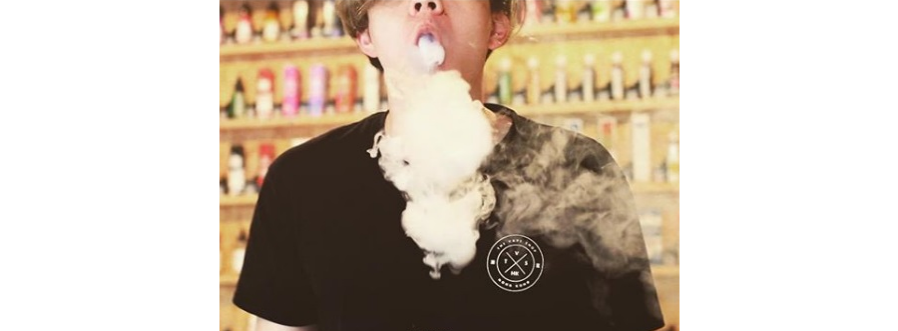
Example of male representation within the "behaviors and practices" domain.

**Figure 10:**
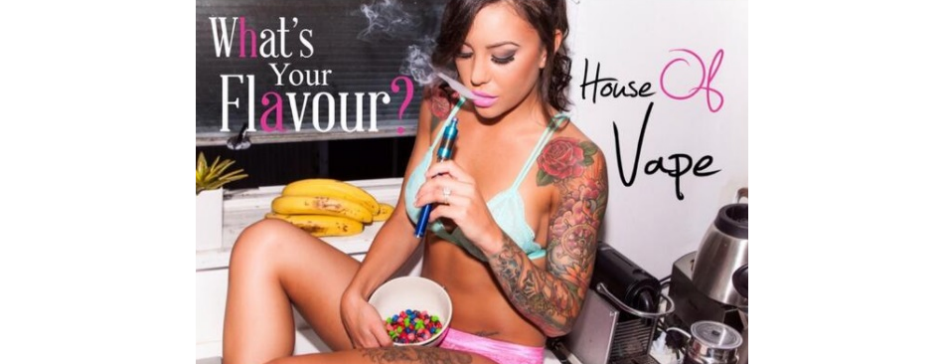
Example of a sexualized image within the "behaviors and practices" domain.

Men were more often represented in selfies (40/55, 73%; *P*<.001), and in posts of people vaping (139/261, 53.3%; *P*<.001) and performing vape tricks (25/43, 58%; *P*<.001; Figure 9) than women (selfies: 12/55, 22%; vaping: 84/261, 32.2%; performing vape tricks: 8/43, 19%). Furthermore, men more frequently posted “hand check/product checks” (98/255, 38.4%; *P*<.001) and posts that indicated a connection with the vape community or vaper identity (199/709, 28.1%; *P*=.05) than women (12/255, 4.7% and 60/709, 8.5%, respectively). A person was present in 18 of the 19 “erotic or sexualized” posts, of which 16 (89%) images contained women scantily dressed and suggestively posed (Figure 10). The remaining 2 images portrayed a man and woman together.

##### Regulation and Advocacy

**Figure 11:**
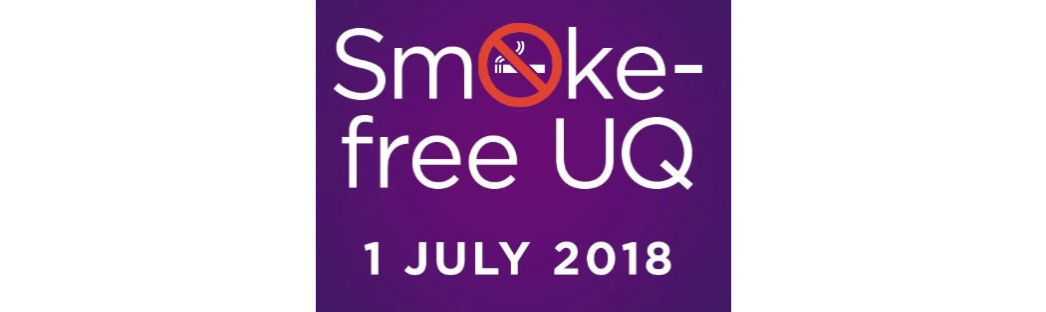
Example of a restrictive policy within the "regulation and advocacy" domain.

**Figure 12:**
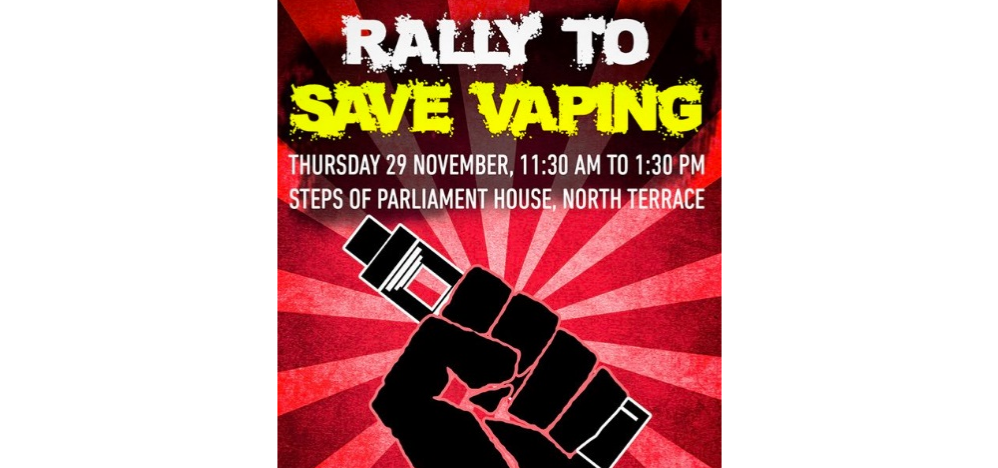
Example of advocacy within the "regulation and advocacy" domain.

E-cigarette regulation and policy were discussed in 10.74% (140/1303) of posts (Table 7). An almost equal proportion of posts was found to be discussing or in favor of liberal (90/1303, 6.91%) versus restrictive (87/1303, 6.68%; Figure 11) e-cigarette policies. Advocacy efforts were encouraged in 4.99% (65/1303) of posts, of which 60% (39/65) supported liberal e-cigarette regulation (Figure 12).

**Table 7:** Frequency statistics for each year corpus and the total sample within the “regulation and advocacy” domain.

Associated codes	2012 (n=12), n (%)	2014 (n=246), n (%)	2016 (n=540), n (%)	2018 (n=505), n (%)	Total (n=1303), n (%)
Regulation or policy	0 (0)	9 (3.7)	43 (8.0)	100 (19.8)	140 (10.7)
Liberal regulation	0 (0)	6 (2.4)	26 (4.8)	58 (11.5)	90 (6.9)
Restrictive regulation	0 (0)	2 (0.8)	27 (5.0)	58 (11.5)	87 (6.7)
Advocacy	0 (0)	3 (1.2)	16 (3.0)	46 (9.1)	65 (5.0)

## Discussion

### Promotional Practices and Strategies

The use of several promotional practices and strategies was documented in this study, namely the promotion of positive perceptions of e-cigarette use, implicit and explicit marketing of e-cigarette products and businesses, and the use of promotional offers (monetary and nonmonetary). These findings are consistent with those reported in a recent systematic review of e-cigarette marketing communication [44] and are known and effective strategies utilized by the tobacco industry for decades [45]. These promotional practices coupled with the ease in which consumers can purchase products online through the click of a link have resulted in the exponential growth of online e-cigarette sales worldwide [46]. Investigations into youth online purchasing have confirmed the ease with which young people can purchase e-cigarette products due to the lack of appropriate age detection processes [47-49].

The promotion of e-liquid flavors through images, detailed flavor descriptions, and appealing product packaging was common and is supported by other social media–based investigations [50,51]. E-cigarette users commonly report the importance of flavored e-cigarette products in facilitating smoking abstinence and enhancement of their vaping experience [52]. Subsequently, e-cigarette manufacturers and retailers have adopted the promotion of flavored e-cigarette products as a major marketing strategy [53]. However, evidence indicates the promotion of flavored e-liquid may be particularly attractive to young people [54] and serve as one of the main reasons for e-cigarette initiation [55]. Furthermore, youth have been found to perceive fruit-flavored e-liquids to be less harmful than tobacco-flavored products [56], and fruit-flavored e-liquids have been linked to greater perceived enjoyment [57].

### Health, Safety and Product Claims

It is not uncommon to find posts on social media claiming e-cigarettes are safer than cigarettes and can be used as a cessation tool, with limited or no validation [35]. Only a very small proportion of posts in this study was accompanied by or depicted a health warning or age restriction, and an increasing proportion of posts was found to be promoting the positive health effects of vaping. Furthermore, a substantial proportion of posts promoted e-cigarettes as a replacement or alternative to cigarettes, similar to that found by Laestadius and colleagues [30]. Risk perception plays an important role in product use decision making, and a commonly cited reason for e-cigarette uptake among adults and young people is the belief that they are less harmful than cigarettes [58-60]. Youth who perceive e-cigarettes as harmless or less harmful than cigarettes are at increased susceptibility of uptake compared to youth with more negative views towards vaping [61,62].

### Behaviors and Practices

A common post found in this study, the “hand check/product check,” is significant because these posts reflect the variety and wide range of vaporizer and e-liquid products and accessories that exist. As vaporizers continue to evolve, with users able to customize and create unique devices, users are increasingly turning to social media to share the products they are using and creating. Similarly, Chu and colleagues [29] found a large proportion of product-based images posted to the social media platform Instagram exhibiting the hashtag #handcheck. The authors expressed concern regarding this increasing trend, as these images act as unpaid marketing of e-cigarette products and viewers may interpret these devices to be commonplace and socially acceptable.

The inclusion of hashtags such as #vapecommunity, #vapelife, #vapenation, and #cloudchaser demonstrate the existence of a vaping identity and community on Twitter, which has also been found in prior vaping-related social media investigations [30,63]. Inclusion of such hashtags may function to create an internalization of social bonding and a vape-related identity [63]. This internalization may help one to define who they are and create their own identity and values within a society that has normalized values and practices. This has led to the formation of unique online and face-to-face “vaper” communities and identities [64,65], which some people are now adopting and associating with rather than the identity of being a “cigarette smoker” or “ex-smoker.” The application of hashtags to social media posts is a form of folksonomy, and the initiating adopters of these electronic tags and subsequent uptake by imitators can be explained by Roger’s Diffusion of Innovation Theory, which seeks to explain how, why, and at what rate new ideas and technology spread [66]. It has therefore been suggested by some that these vaping-related discussions may be occurring within some networks as an “echo chamber,” whereby the ideas and beliefs of those within the network are strengthened, resulting in the normalization of vaping within these communities [63]. Research examining Australian Twitter users using network analysis methods could provide an Australian perspective on this hypothesis. Further, research that examines how nicotine addiction is represented on social media may assist to understand evolving perceptions of addiction and identity.

### Implications for Policy and Research

This investigation demonstrates that a number of Australian Twitter users are purposefully (commercial) and also inadvertently (through posts by vapers) promoting the use of e-cigarettes. Twitter has a “paid” advertising policy prohibiting the promotion of tobacco products, accessories, and branding (including e-cigarettes) [67]. The policy, however, does not relate to individual account holder’s content, fan pages, or groups. The boundaries between owned, paid, earned, and shared content have become increasingly more blurred [68], with evidence suggesting influencers are being used to circumvent social media policies [69,70]. In the absence of regulations controlling online promotions and formal gateways restricting access to content, posts on social media platforms such as Twitter can reach and potentially influence both e-cigarette users and nonusers alike [51]. Exploring opportunities to further restrict the commercial promotion of these devices (ie, unpaid promotion from commercial accounts) on Twitter and other social media platforms is required, and working with social media platforms to voluntarily employ these restrictions is one possible solution [71].

This study found the proportion of posts specifically promoting e-cigarette products for purchase decreased in 2018 (Multimedia Appendix 1), although this correlates with a relative decline in Twitter use by Australians in comparison to other larger and growing platforms. Due to the increased popularity of Instagram over recent years, and more recently TikTok, it would be valuable to investigate e-cigarette–related promotional content posted to these platforms. Instagram and TikTok are primarily photo and video-sharing social networking services; therefore, these platforms may be more desirable and more highly accessed than Twitter to share this type of content.

A product for therapeutic use, such as smoking cessation or alleviation of nicotine withdrawal, must be registered with the Therapeutic Goods Administration to be sold lawfully in Australia [2]. At present, no heated tobacco nor nicotine vaporizer has been approved by the Therapeutic Goods Administration and therefore should not be promoted as a smoking cessation product. Continued monitoring of Australian e-cigarette retailers to ensure misleading health and smoking cessation claims are not being made is therefore important so as not to contribute further to the confusion regarding e-cigarette safety and efficacy.

### Limitations

Several limitations need to be considered when interpreting the results of this study. This study reflects data from one social media platform, Twitter, as its data are mostly public and easily accessible to researchers, whereas some other social media platforms are not as readily accessible [72]. However, the TrISMA infrastructure makes Australian-specific historical Twitter data accessible in a way most other social media platforms do not. This is not an indication that other social media platforms are not spaces where e-cigarettes are discussed by Australians, but only that these activities are not always as visible to researchers. The search strategy included several popular terms used to describe e-cigarettes and vaping practices; however, emerging and variations of slang terms may have been overlooked. The investigation focused only on tweets that included an image. Therefore, these results may not be reflective of all tweets by Australian users. Lastly, we relied on TrISMA’s programmed bot filtering processes occurring at the level of the user before tweets were collected to remove questionable accounts. Future studies examining Twitter data are encouraged to apply denoising techniques after data collection [73].

### Conclusions

Despite Australia’s cautious approach toward e-cigarettes and the limited evidence supporting e-cigarettes as an efficacious smoking cessation aid, it is evident that there is a concerted effort by some Twitter users to promote these devices as a harmless, health-conducive, smoking cessation product. Further, Twitter is being used in an attempt to circumvent Australian regulation and advocate for a liberal approach to personal vaporizers. The borderless nature of social media presents a clear challenge for enforcing Article 13 of the WHO FCTC. Evidence suggests a relationship exists between e-cigarette advertising exposure and uptake, and social media is now being used to generate favorable attitudes towards vaporizer products. As “digital media” consumption has increased, content that was previously inaccessible due to conventional advertising regulations, such as tobacco advertising, is now visible, and traditional tobacco control regulations are no longer adequate. The internet is the perfect platform to promote e-cigarettes and novel nicotine products, even in a highly regulated country such as Australia. Countering the advertising and promotion of these products is a public health challenge that will require cross-border cooperation with other WHO FCTC parties.

## Appendix

Multimedia Appendix 1 Coding framework.

## Abbreviations

e-cigarette: electronic cigarette
GIF: graphic interchange format
TrISMA: Tracking Infrastructure for Social Media Analysis
WHO FCTC: World Health Organization Framework Convention on Tobacco Control
