# Supplementary material for: E-Cigarette Promotion on Twitter in Australia: Content Analysis of Tweets
Source: JMIR Public Health Surveill. 2020 Nov 5;6(4):e15577. doi: 10.2196/15577 (PMC7677022; doi:10.2196/15577)
Supplement: Multimedia Appendix 1 [file publichealth_v6i4e15577_app1.docx]

| **Codes and definitions** | **Coding rule** |
| --- | --- |
| **Product visible**  1 = e-cigarette or other vaping device  2 = e-cigarette and another vape/tobacco product  3 = vape accessory (e.g. tank)  4 = vape liquid (e-liquid)  5 = vape liquid and another vape/tobacco product  6 = showcase in a retail store  7 = tobacco product  8 = none | Are any of the following products visible in the image? |
| **Product placement**  1 = overt (i.e. shown openly, or plainly apparent, and is the main focus of the image)  2 = covert (i.e. not openly displayed, or hidden, and is not the main focus of the image)  3 = N/A (if no product visible) | How is the vape/tobacco product placed in the image? |
| **Product brand or logo visible**  1 = yes  2 = no  3 = N/A (if no product visible) | Is a brand or logo visible on the vape/tobacco product? |
| **Product brand or logo visible anywhere**  1 = yes  2 = no | Is a vape/tobacco brand or logo visible anywhere in the image? |
| **E-liquid flavour described**  1 = yes  2 = no  3 = N/A (if no depiction of an e-liquid product, or does not describe in text) | Does the post indicate the flavour of e-liquid in the image or in the text? |
| **Nicotine level**  1 = nicotine level in milligram  2 = nicotine free  3 = multiple products, nicotine level and nicotine free  4 = no level  5 = N/A (if there is no depiction of an e-liquid product) | Is the nicotine level displayed on either the e-liquid bottle or packaging or stated in the text? |
| **Health or age warning visible**  1 = yes  2 = no | Is a health or age warning visible in the image or stated in the text? |
| **People visible**  1 = yes  2 = no | Are there people, or part thereof (e.g. hand), visible in the image? |
| **Type of people visible**  1 = everyday person  2 = model (i.e. stylised image, or image taken for promotional or media purposes)  3 = celebrity  4 = health professional/academic  5 = other  6 = multiple types  8 = N/A (if no person/face visible) | What type of people are visible in the image? Only code for when a face is visible. |
| **Gender of people visible**  1 = female  2 = male  3 = both male and female  4 = cannot determine  5 = N/A (if no people visible) | What gender are the people that are visible in the image? |
| **Age of people visible**  1 = less than 18 years  2 = 18 years or older  5 = mixed age groups  6 = N/A (if no person/face is visible) | What age are the people visible in the image? Only code for when a face is visible. |
| **Vapour present**  1 = yes  2 = no | Is vapour present in the image? |
| **Person vaping**  1 = yes  2 = no | Are people vaping in the image or described in the text? |
| **Vape play**  1 = yes  2 = no | Are people performing vape tricks or cloud chasing in the image or described in the text? Cloud chasing is the activity of blowing large clouds of vapour using a vaping device |
| **Setting of product**  1 = indoors (including cars)  2 = outdoors  3 = N/A (if no vape product is displayed or being used) | What setting is the vape product displayed or being used in the image or described in the text? |
| **Association with another product**  1 = cannabis (including hemp)  2 = alcohol  3 = none | Are any of the following substances displayed in the image or described in the text and being associated with vaping? |
| **Anti or critical of vaping**  1 = yes  2 = no | Is the post anti or critical of vaping? |
| **Product review**  1 = yes  2 = no | Is the post alerting viewers to a product review? |
| **Promoting vape product for purchase**  1 = yes  2 = no | Is the post promoting a vape product for purchase? Would usually be from a retailer, manufacturer or product reviewer (does not including posts which are giving away items) |
| **Promotional offer**  1 = monetary (any promotional deal that saves you money, lowers the cost of a purchase or changes the cost of the purchase. e.g. coupons, refunds, rebates, two for one deals, and cents-off promotions)  2 = non-monetary (any promotional deal that does not lower the cost of a purchase. e.g. contests, giveaways, sweepstakes, free shipping or free gift with purchase)  3 = both  4 = none | Is the post providing monetary or non-monetary offers? |
| **Sale notice**  1 = yes  2 = no | Does the post introduce customers to the idea that a discount is being offered without actually stating what the savings are? |
| **Business listing**  1 = yes  2 = no | Is the post a business listing, or a notice advertising vape shops, e-cigarette merchants, brands or online vape groups? The post may also aim to recruit new followers or members by way of retweeting, liking or tagging. |
| **Quit smoking**  1 = yes  2 = no | Does the post promote vaping as a means to quit smoking or as an alternative to tobacco cigarettes? Includes related hashtags. |
| **Health**  1 = positive health  2 = negative health  3 = no | Does the post reference positive or negative health effects of vaping? |
| **Safety**  1 = yes  2 = no | Does the post reference how to use/build e-cigarettes or handle nicotine safely, battery safety, or safety of e-liquid ingredients? |
| **Public health**  1 = yes  2 = no | Does the post reference public health professionals, journals or organisations, or net public health gains or losses concerning e-cigarettes? |
| **Youth vaping**  1 = yes  2 = no | Does the post refer to youth vaping? |
| **Regulation or policy**  1 = yes  2 = no | Does the post reference regulation or policy in regards to vaping, whether positive, negative or neutral? |
| **Liberal regulation**  1 = yes  2 = no | Does the post support less restrictive e-cigarette regulation/policy, or refer to liberal regulation/policy which has been proposed or enacted? |
| **Restrictive regulation**  1 = yes  2 = no | Does the post support restrictive e-cigarette regulation/policy, or refer to restrictive regulation/policy which has been proposed or enacted? |
| **Advocacy**  1 = yes  2 = no | Does the post reference advocacy activities or efforts? |
| **Building/DIY**  1 = yes  2 = no | Does the post reference modifying or ‘building’ vape products/accessories? |
| **Cartoon**  1 = yes  2 = no | Does the image contain animations or cartoons? This can include on vape product packaging. |
| **Meme**  1 = yes  2 = no | Is the post a meme? A meme is an image accompanied by a piece of text which is typically humorous. Memes can be copied and spread rapidly by Internet users, often with slight variations. |
| **Selfie**  1 = yes  2 = no | Is the image a self-portrait style image, also known as a ‘selfie’? |
| **Hand check/product check**  1 = yes  2 = no | Is the image of a vape product in a person’s hand or is the post showing what product the poster is currently using? |
| **Erotic or sexualised**  1 = yes  2 = no | Does the image portray scantily clad people or someone in a suggestive pose? |
| **Identity or community**  1 = yes  2 = no | Does the post convey a vaping social identity or shared community affiliation, this includes hashtags (i.e. #vapecommunity, #vapelife) |
| **Malfunction**  1 = yes  2 = no | Does the post reference e-cigarette devices malfunctioning? |
